# Melatonin: Both a Messenger of Darkness and a Participant in the Cellular Actions of Non-Visible Solar Radiation of Near Infrared Light

**DOI:** 10.3390/biology12010089

**Published:** 2023-01-06

**Authors:** Dun-Xian Tan, Russel J. Reiter, Scott Zimmerman, Ruediger Hardeland

**Affiliations:** 1 Department of Cell Systems and Anatomy, UT Health San Antonio, Long School of Medicine, San Antonio, TX 78229, USA; 2 Silas, Inc., Basking Ridge, NJ 07920, USA; 3 Johann Friedric Blumenbach Institute of Zoology and Anthropology, University of Göttingen, D-37073 Göttingen, Germany

**Keywords:** light, near-infrared radiation, mitochondria, melatonin synthesis, microbiota, pineal gland, cerebrospinal fluid, circadian rhythm

## Abstract

**Simple Summary:**

Environmental light exposure is an important factor for human health that impacts the biological clock of organisms. Melatonin usually serves as a chemical expression of darkness since light exposure suppresses its synthesis. The low level of melatonin is associated with a spectrum of disorders since melatonin is a potent endogenous antioxidant; therefore, individuals should avoid visible light exposure at night. However, evidence also shows that near infrared radiation (NIR), which occupies a major portion of the wavelengths of sunlight, promotes melatonin production and the beneficial effects of sun baths or photobiomodulation therapy may be, at least, partially mediated by the increased local melatonin production induced by NIR. Unlike visible light, NIR can penetrate deep into the human body including the muscle, brain, and even the bones, and its effects on human biology remain to be clarified. Thus, the avoidance of light at night and exposure to sun during the day are equally important to improve melatonin production and human well-being.

**Abstract:**

Throughout the history of melatonin research, almost exclusive focus has been on nocturnally-generated pineal melatonin production, which accounts for its circadian rhythm in the blood and cerebrospinal fluid; these light/dark melatonin cycles drive the daily and seasonal photoperiodic alterations in organismal physiology. Because pineal melatonin is produced and secreted primarily at night, it is referred to as the chemical expression of darkness. The importance of the other sources of melatonin has almost been ignored. Based on current evidence, there are at least four sources of melatonin in vertebrates that contribute to the whole-body melatonin pool. These include melatonin produced by (1) the pineal gland; (2) extrapineal cells, tissues, and organs; (3) the microbiota of the skin, mouth, nose, digestive tract, and vagina as well as (4) melatonin present in the diet. These multiple sources of melatonin exhibit differentially regulated mechanisms for its synthesis. Visible light striking the retina or an intense physical stimulus can suppress nocturnal pineal melatonin levels; in contrast, there are examples where extrapineal melatonin levels are increased during heavy exercise in daylight, which contains the whole range of NIR radiation. The cumulative impact of all cells producing augmented extrapineal melatonin is sufficient to elevate sweat concentrations, and potentially, if the exposure is sustained, to also increasing the circulating values. The transient increases in sweat and plasma melatonin support the premise that extrapineal melatonin has a production capacity that exceeds by far what can be produced by the pineal gland, and is used to maintain intercellular homeostasis and responds to rapid changes in ROS density. The potential regulatory mechanisms of near infrared light (NIR) on melatonin synthesis are discussed in detail herein. Combined with the discovery of high levels of melanopsin in most fat cells and their response to light further calls into question pineal centric theories. While the regulatory processes related to microbiota-derived melatonin are currently unknown, there does seem to be crosstalk between melatonin derived from the host and that originating from microbiota.

## 1. Introduction

Melatonin is a derivative of the essential amino acid, tryptophan. It was first isolated from the bovine pineal gland and its chemical structure was identified as the methoxyacetyltryptamine [1]. It is referred to be an indolamine by most of researchers, but more correctly, its chemical structure is that of an amide. It is a phylogenic old molecule since, during evolution, it is believed to have emerged in primitive bacteria, probably 2.5 billion years ago [2]. Melatonin is likely to be present in all three life domains including bacteria, archaea, and eukaryotes. Its synthesis in bacteria and eukaryotes has been well-documented and melatonin has been detected in these species by a variety of methods. In archaea, the presence of melatonin was predicted [3], with this prediction being confirmed by a recent observation in which Back et al. [4] reported the presence of a functionally active serotonin N-acetyltransferase (SNAT) (or arylalkylamine N-acetyltransferase (AANAT) in an archaeon (i.e., of the penultimate enzyme in the melatonin biosynthetic pathway that catalyzes the formation of N-acetylserotonin from serotonin). When this archaeal *AANAT* gene was cloned and expressed in rice, it resulted in an increased melatonin content. Although the purified enzyme exhibited SNAT activity in vitro [4], additional evidence of the presence of melatonin in archaea would be required using GCMS technology.

Melatonin is a multifunctional antioxidant with several unique characteristics that distinguish it from the classic antioxidants. For example, one molecule of melatonin may scavenge up to 10 oxygen reactive species (ROS)/or nitrogen reactive species (NOS) [5], whereas a classic antioxidant such as vitamin C only scavenges one or two of such reactants. The mechanism for this is that the metabolites generated by melatonin interaction with ROS also possess an antioxidant capacity. This reaction of melatonin with its second, tertiary metabolites continuously scavenging ROS, is referred to as a cascade reaction of melatonin toward free radicals [6]. Moreover, melatonin has little prooxidative activity, which is not the case for some classic antioxidants. In addition, melatonin, as an amphiphilic (both lipid and water soluble) molecule, serves as an antioxidant in both the cell membrane and in all intracellular compartments. Due to these superior features over classic antioxidants, melatonin is probably one of the earliest antioxidants that evolved and it still functions as the first line of defense to protect all molecules from oxidative stress. The protective effects of melatonin against oxidative stress have been documented from unicellular organisms, plants to vertebrates [7,8,9,10]. The primary function of melatonin is as an antioxidant with other functions being acquired at different stages of evolution [2]. These acquired functions include the conveyance of the message of darkness [11], the regulation of reproduction in photoperiodic animals [12], sleep promotion in diurnal animals [13,14], modulation of energy metabolism [15], anti-inflammation [16], and oncostatic activity [17].

These multiple functions make melatonin a versatile and important molecule, with its deficiency being associated with a variety of disorders including neurodegenerative diseases, heart diseases, metabolic disorders, osteoporosis, cancer, and even aging [18,19,20,21,22,23]. It is well-documented that the serum and cerebrospinal melatonin levels exhibit a day/light rhythm with high levels at night and low baseline values during the day in all vertebrates [24]; these rhythms are exclusively due to the release of melatonin from the pineal gland at night. However, in addition to those of pineal origin, organisms have three additional sources of melatonin. Extrapineal sources of melatonin include the mitochondrial melatonin potentially produced in all other cells, tissues, and organs and that from microbiota as well as from diet. Indeed, Zhao et al. [25] speculated that the pineal gland accounts for less than 5% of the total melatonin produced in mammals. Unlike pineal melatonin, that portion synthesized in these elements is mostly not released in substantial quantities into the blood or the CSF, but, rather, is used in the cell/bacterium in which it was produced. This is generally referred to as the non-releasable pool of melatonin [26]. However, it is important to distinguish between cells and organs that generally do not release melatonin and others that allow for high rates of release on a conditional basis. An impressive example for the latter possibility is the gastrointestinal tract. Under basal conditions, melatonin is only poorly released from this extrapineal source, but upon stimulation by tryptophan supply, high amounts, exceeding by far those secreted by the pineal gland, enter the circulation, as shown in chicken and rats [27] as well as in humans [28]. These effects were observed in humans after the infusion of 5 g tryptophan in both the morning and evening [28]. Even the administration at 8:00 a.m. resulted in levels about 2.5 times higher than the normal circadian maximum observed at night. Lower tryptophan amounts such as those present in the normal food have been discussed as one cause of postprandial melatonin release [29]. The example of the gastrointestinal tract also shows that the size of the extrapineal source has to be considered when discussing its contribution to circulating levels. Due to its size, the gastrointestinal tract contains approximately 400-fold amounts of melatonin relative to those present in the pineal gland and in the circulation [27,30]. Therefore, the potential for releasing high quantities is correspondingly high. The precise mechanism by which tryptophan stimulates the release from the melatonin-synthesizing enterochromaffin cells requires further elucidation. Moreover, it has not yet been studied in detail whether the intestinal microbiome also responds to elevated tryptophan and may therefore contribute to the observed elevations. However, the microbiome should not be suspected to be responsible for the complete increase in circulating melatonin because this would be at variance with substantial differences between tryptophan-induced elevations in the morning and the evening [28]. The relevance of the size of an extrapineal source of melatonin is not restricted to the gastrointestinal tract. Another melatonin-producing organ of considerable size is the skin [31,32], although it does not seem to release similarly high quantities, as observed with the gastrointestinal system. However, the skin will be shown to be involved in responses that are central to the topic of this article.

In the current review, we discuss the biological activities and the potential regulatory mechanisms of melatonin synthesized at different sites. Moreover, one focus will be on the recently-discovered role of the effects of NIR radiation on extrapineal melatonin regulation. The rationale is the high penetrability of NIR into the body and the fact that NIR is currently of great medical interest as it is being used to treat a number of medical conditions that may also relate to melatonin (see Section 4).

## 2. Melatonin Synthesis in Mitochondria and Its Biological Significance

**Figure 1:**
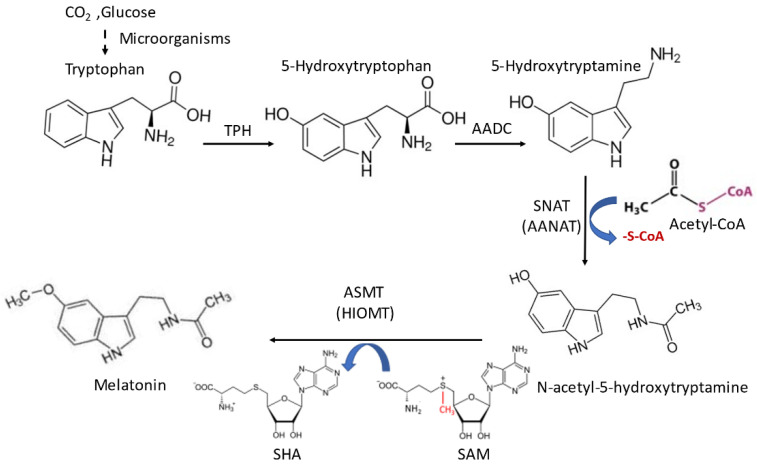
The classic melatonin biosynthetic pathway in microorganisms and animals. Melatonin synthesis occurs primarily in the mitochondria of the cells. TPH: tryptophan hydroxylase, AADC: aromatic amino acid decarboxylase, SNAT (AANAT): aralkylamine N-acetyltransferase, ASMT (HIOMT): N-acetylserotonin O-methyltransferase (hydroxyindole-O-methyltransferase), SAM: S-adenosyl methionine, SHA: S-adenosyl homocysteine.

As above-mentioned, melatonin is a derivative of tryptophan. In animals, tryptophan is the initial precursor of melatonin. Tryptophan has to be taken up from food because animals lack the shikimic acid pathway. Via four consecutively enzymatic steps including hydroxylation, decarboxylation, acetylation, and methoxylation, tryptophan is converted to melatonin (Figure 1). However, in microorganisms or plants that possess the capability of synthesizing aromatic amino acids via the shikimic acid pathway, the initial precursor does not have to be tryptophan, but can be glucose or even carbon dioxide [33]. This may be a reason why microorganisms and plants generate much larger amounts of melatonin than animals do since their melatonin synthesis capacity is not limited by the availability of tryptophan [34,35]. Generally, the last step of melatonin synthesis is methoxylation by acetylserotonin methyltransferase (ASMT), but under some conditions, the acetylation by AANAT may serve as the last step of melatonin synthesis [36]. This has been often observed in microorganisms and plants. Under stressful conditions, the major melatonin synthetic pathway in plants switches to acetylation as the final step to dramatically improve melatonin production [37]. With the expectation that a similar association occurs in animals, melatonin may provide uncommonly elevated protection against ROS during high stress conditions (exercise, solar exposure, heat exhaustion, digestion, and anxiety attacks).

It was originally widely accepted that melatonin was synthesized in the cytosol [38], even though there was no solid data to support this opinion. The majority of the melatonin synthetic studies were carried out in the supernatants of the tissue or cell homogenates. The homogenates, if not going on to further purification, contain not only the cytosol, but also all the other cellular organelles. Even when the supernatants were purified, the contents of the broken organelles could still have leaked into the supernatants and masked the study results. This is particularly valid for mitochondria, especially when existing in a state of extensive fusion. Based on the high AANAT staining in the mitochondria of pinealocytes [39] and the high concentration of melatonin measured in the purified mitochondria of hepatocytes, it was hypothesized that melatonin was synthesized in the mitochondria [40]. Moreover, it was also noted that the taxon containing the phylogenetic precursors of mitochondria, alpha-proteobacteria, possessed the capacity to synthesize melatonin. Thus, mitochondria might have retained this capacity to defend against oxidative stress in eukaryotic cells. This hypothesis has been proven in both animals and plants including where the isolated mitochondria from mouse oocytes or apple tree leaves efficiently synthesize melatonin [32,33]. The SNAT and ASMT proteins as well as their respective mRNAs are localized in the mitochondria of plants and various animal cells [35,36]. Recently, Isola et al. [37], with the use of immunohistochemistry and transmission electron microscopy, identified melatonin in the mitochondria of human salivary gland cells, suggesting its synthesis in this organelle.

Perhaps the most convincing study related to melatonin synthesis in mitochondria is that of Suofu et al. [41]. They detected both AANAT and ASMT proteins in brain non-synaptosomal mitochondria, whereas these enzymes were virtually absent in the cytosol. In pinealocytes, as expected, the expression of AANAT protein exhibited a circadian rhythm, whereas no rhythm in AANAT expression was demonstrable in non-synaptosomal mitochondria from neurons, indicating a profound functional difference between these two cell types. Thus, the continuously generated melatonin in the mitochondria of neurons likely provides constant protection to these cells against oxidative stress, whereas melatonin rhythmically produced in pinealocytes serves the release into blood and CSF to convey a signal of photoperiodic alterations. The available evidence strongly indicates that melatonin is synthesized in mitochondria. That being the case, the findings suggest that most cells synthesize melatonin. Judging from the available data, this is the case since the expression of melatonin synthetic enzymes, AANAT and ASMT, have been identified in all cells tested including neurons, glia, astrocytes, thymus, heart, kidney, spleen, lymphocytes, oocytes, testes, placenta, skin, gut, etc. [42,43,44,45,46,47,48,49].

Melatonin synthesis in mitochondria is of great biological significance. First, mitochondria are a major source of ROS and some RNS [50]. Thus, the locally synthesized melatonin would provide on-site protection. Second, acetyl coenzyme A is synthesized in mitochondria and is an essential substrate of AANAT for melatonin synthesis (Figure 1). Its concentration in mitochondria is in the range of K_m_ of AANAT and this makes melatonin synthesis in mitochondria much more efficient compared to other cellular compartments [51]. Melatonin’s biosynthesis requires healthy mitochondria to provide this substrate. In dysfunctional mitochondria, melatonin production is compromised. Under pathological conditions such as in cancer, the cancer cell switches mitochondrial glucose oxidation to cytosolic glycolysis (i.e., the Warburg effect [52,53]), and therefore less acetyl coenzyme A is generated in the mitochondria. Thus, the mitochondria of cancer cells have only about half the melatonin levels in the mitochondria compared to normal cells [54,55], consistent with the depressed levels of melatonin in cancer patients [56,57,58].

Mitochondrial DNA has a much higher mutation rate than nuclear DNA. During aging, the accumulation of the mutated mitochondrial genome forms what is referred to as mitochondrial genome heteroplasmy [59]. When this heteroplasmy reaches a certain threshold, the mitochondrial function declines [60], as does, presumably, their melatonin production. This may be a major reason for the reduced melatonin production with aging in many species. Another phenomenon relative to mitochondria is the so-called mother’s curse, which hypothesizes that the lower mitochondrial function in males compared to females is due to the maternally inherited features of these organelles [61,62]. Therefore, there may be a potential melatonin synthetic bias in favor of the female. This melatonin synthetic dimorphism may explain the higher resistance of females to some infectious diseases such as COVID-19, or the relatively slower aging processes of females than males [22,63,64]. Furthermore, melatonin deficiency causes mitochondrial dysfunction. For example, knockdown of the *AANAT* gene in murine embryonic cells caused an abnormal mitochondrial distribution, significantly decreased mtDNA copy numbers, elevated base mutations in the D-loop of replicating mtDNA. and retarded embryonic development compared to the wild type [65]. An additional effect of *AANAT* knockdown with numerous consequences to the regulation of gene expression concerns a suppression of tet methylcytosine dioxygenase 2 (Tet2), which results in reduced DNA demethylation. AANAT knockout mice exhibited, in the brain and primary cerebro-cortical neurons, increased mitochondrial oxidative stress and reduced mitochondrial membrane potential, with higher mtDNA release into the cytoplasm. The cytosolic appearance of mtDNA caused activation of the cGAS/STING/IRF3 pathway, which resulted in elevated inflammatory cytokine production [66]. Interestingly, transgenic Huntington’s disease mice carrying a mutant huntingtin (mHTT) protein that binds to the TIM23 mitochondrial protein import complex showed changes of oxidative damage in the cortex and striatum reminiscent of those found in AANAT deficient animals [67]. The mitochondrial alterations caused by melatonin deficiency, or mutant mHTT, in the cells or in the animals above-mentioned are rescued by melatonin application. The mitochondrial function and melatonin production reciprocally impact each other positively or negatively. In the negative aspect, the mitochondrial malfunction results in low melatonin production, which then further manifests the mitochondrial dysfunction to become a vicious cycle. Melatonin supplementation could break this vicious cycle and improve mitochondrial function. This has been demonstrated in a variety of mitochondrial disorders including ischemia/reperfusion, heart diseases, neurodegenerative diseases (Alzheimer’s, Parkinson’s, and Huntington’s diseases), and metabolic disorders (type 2 diabetes, obesity, fatty liver disease), in which melatonin intervention leads to improved outcomes in these disorders [68,69,70,71,72,73,74].

Several mechanisms have been identified by which melatonin improves mitochondrial function; only a few of them are mentioned here.

(1) Melatonin promotes the activity of pyruvate dehydrogenase (PDH) to enhance mitochondrial uptake of pyruvate, which increases the production of acetyl coenzyme A, a necessary co-factor for melatonin synthesis. Thus, melatonin has the capacity to switch the so-called Warburg’s effect to mitochondrial oxidative metabolism in cells [75,76,77].
(2) Melatonin increases the activities of mitochondrial complexes 1 and 3 to accelerate electron transport and also reduces the electron leakage and lowers the associated oxidative stress [78,79].
(3) Melatonin upregulates the expression and activity of mitochondrial uncoupling proteins 1/2/3 to balance the potential overshoot of mitochondrial membrane potential and lowers the free radical generation [80,81,82].
(4) Melatonin supports the transfer of functionally active mitochondria from healthy to injured cells, thereby rescuing the energy metabolism of the recipient cells [83]. The transfer of mitochondria is achieved either by tunneling nanotubes that develop between healthy and injured cells or via exosomes, whose cargo comprises diverse cellular materials including regulatory RNAs and organelles such as mitochondria [84].
(5) Melatonin regulates mitochondrial dynamics. This includes melatonin’s promotion of mitochondrial biogenesis in both stem cells and postmitotic cells. Under most conditions, melatonin increases mitochondrial fusion, inhibits their fission, and enhances mitophagy [85,86,87,88], whereas the reverse only occurs in tumor cells [89]. In nontumor cells, it downregulates the genes involved in mitochondrial fission (DRP1, hFis1, MIEF2, MFF) and mitophagy (PINK, BNip3, NIX) to maintain mitochondrial homeostasis.
(6) Melatonin inhibits the mitochondrial permeability transition pore (mtPTP) opening to preserve the mitochondrial membrane potential and maintain functionally intact mitochondria [90,91]. The activity of melatonin in influencing mitochondrial physiology may be in part receptor mediated, since the membrane melatonin receptor 1 (MT1) is not restricted to the plasma membrane, but also located on the mitochondrial membrane [41]. The signal transduction processes by which melatonin modulates mitochondrial biogenesis is via the MT1/SIRT1/PGC-1α/NRF2/PPAR-γ pathway [92].

Melatonin synthesis in mitochondria does not per se exclude melatonin synthesis in cytosol. For example, erythrocytes, which are devoid of mitochondria, are known to synthesize some amounts of melatonin [93]. However, the involvement of AANAT and ASMT has not been demonstrated and the formation via unspecific N-acetyltransferases and O-methyltransferases cannot yet be excluded. Judging from the criterion of substrate availability, cytosolic melatonin synthesis would be far less efficient than that in mitochondria and may be less biologically significant.

## 3. Regulation of the Visible Light (Blue Wavelength) on Cerebrospinal Fluid (CSF) and Serum Melatonin Circadian Rhythms: Melatonin Serving as the Chemical Expression of Darkness

The primary function of melatonin serves as an antioxidant in all organisms while other functions of melatonin were acquired throughout evolution. One of the acquired important functions of melatonin is that it serves as the chemical expression of darkness. In this capacity, melatonin regulates physiological adjustments made in response to the changing photoperiodic environment, helping them to cope with day/night or seasonal changes. Unicellular organisms such as bacteria and microalgae directly perceive photoperiodic information and there is no requirement for them to transduce this information. When unicellular organisms evolved into larger multicellular organisms, in which the visible light did not penetrate multicellular barriers, melatonin was selected as the signaling molecule. The rationale for melatonin being selected as the signaling molecule to identify night and day perhaps stemmed from the fact that photosynthetic bacteria and algae already had high levels of melatonin during darkness and lower levels during the day [94,95]. In these organisms, melatonin is synthesized in high quantities throughout the daily cycle, allowing for efficient protection against ROS. However, during the day, the photosynthetic activity of the organisms generates large amounts of ROS, and melatonin, as the first line antioxidant, is rapidly consumed while, during darkness without photosynthesis, less ROS is produced: thus, the lower consumption of melatonin at night might already result in a rhythm with elevated nocturnal melatonin compared to that of the photophase. The differential consumption may have made melatonin particularly suitable for its use as a mediator of scotophase (dark phase). The next step, which already took place in the unicellular stage, was the coupling of melatonin production to circadian oscillators [96]. Whether or not a pre-existing circadian melatonin rhythm is phylogenetically directly transmitted to multicellular organisms is unknown. The profound mechanistic differences between circadian oscillators of bacteria, dinoflagellates, metazoans, and plants speaks against this possibility. Specific to vertebrates, a special organ, the pineal gland, evolved to produce and release melatonin during night as the signal of darkness. The pineal gland was initially directly photosensitive, but this property was lost in mammals, presumably during the subterraneous period in the Mesozoic, also known as the nocturnal bottleneck [97]. To maintain the functional relationship between photoperiodic input and pineal melatonin secretion, a neural connection between the gland and the lateral eyes evolved, which also included the suprachiasmatic nucleus (i.e., circadian master clock). Therefore, the pinealocytes received photoperiodic information adjusted to circadian timing. The pineal gland is juxtaposed to the third ventricle near the center of the brain. Importantly, it has a very rich blood supply. The minimum rate of pineal blood flow per gram tissue exceeds that of most organs and is surpassed only by that of the kidney [98]. It was calculated that the rate of pineal blood flow is 16 times higher than that of the average blood flow per gram tissue in rats [99]. Such high blood flow indicates that the pineal gland may participate in cerebrospinal fluid (CSF) production and therefore, it can easily distribute its major secretory product of melatonin to the entire brain via CSF circulation [100,101]. The parenchymal cells of the pineal gland mainly include pinealocytes, microglia, and astrocytes. The microglia and astrocytes also synthesize melatonin [102], but this does not contribute to the CSF and blood melatonin circadian rhythm since their melatonin production is not rhythmic [103]. These cells can be considered as the supporting cells for normal functions of pinealocytes. Pinealocytes are probably differentiated from Pax6-expresssing neuroepithelial cells [104]. Characteristically, the pinealocytes contain large numbers of mitochondria compared to neurons or other cells. This makes pinealocytes capable synthesizing melatonin at their maximum capacity when needed. The structure and dynamics including the fusion as well as fission of mitochondria in pinealeocytes are altered by photoperiodic alterations. For example, many mitochondria are swollen, with rarified matrix and reduced numbers of cristae under constant light exposure [105]. Thus, even before biochemical studies were performed, it was clear that the prevailing light:dark environment impacted pineal physiology [106].

Many studies have proven that the melatonin synthetic activity of the pinealocytes is regulated by the natural dark/light cycle. While in some vertebrate pineal tissue, rudimentary photoreceptors remain as the mammalian pineal gland is connected to the external photoperiodic environment due to it connection with the eyes. Therefore, a means of transferring the light signal from the eyes to the pineal must exist, and there is also a requirement for the transduction of the neural message into a hormonal signal. The neural pathway between the eyes and the pineal involves the suprachiasmatic nucleus (SCN)–melatonin loop [99]. Briefly, during darkness, the efferent fibers that enter the pineal gland from the superior cervical ganglia (SCG) constantly release norepinephrine onto pinealocytes to stimulate melatonin synthesis. This results in the nightly surge of melatonin production in the pineal gland. The pineal gland has no mechanism of melatonin storage and most of the melatonin is immediately released via the pineal recess into the CSF of the third ventricle of the brain where melatonin circulates in the CSF and protects the brain from oxidative stress [107]. At the same time, some melatonin is released or leaks into the capillaries in the pineal gland. The nocturnal CSF melatonin levels are much higher than the blood levels at night [108], It has been reported that the melatonin level in the CSF is higher in the CSF of third ventricle close to the pineal recess than in other areas of the ventricles, indicating that melatonin is directly discharged into the pineal recess [109,110]. During the day, the melanopsin-containing retinal ganglion cells (MRGC) in the eyes respond especially to blue wavelengths of light with the signal being sent to the SCN via the retino-hypothalamic tract (RHT). SCN relays the signal to the paraventricular nucleus (PVN), which transfers the signal to the intermediolateral cell column (IMCC) and further to the SCG, where the neuronal input signal of light suppresses the activity of the postganglionic sympathetic fibers and diminishes the NE release onto the pinealocytes, resulting in the reduced melatonin production.

The regulatory mechanisms of NE on melatonin production in pinealocytes are quite unique and may differ from that in extrapineal tissues. In rodents, NE acts on both the β_1_ and α_1_ adrenergic receptors in the pinealocyte membrane. The β_1_ adrenergic receptor is a G-protein coupled receptor associated with the Gs heterotrimeric subunit, which couples transmembrane adenylyl cyclase (tmAC). Stimulation of the β_1_ receptor of pinealocytes by NE activates the tmAC and increases the intracellular cAMP concentration, which activates the cAMP-dependent protein kinase A (PKA). In rodents, the NE/β1/cAMP/PKA pathway leads to phosphorylation of the transcription factor of cyclic AMP response element (CRE)–binding protein (CREB) in the nuclei of pinealocytes. The phosphorylated CREB (pCREB) enhances the transcription of *AANAT*, which leads to a dramatical increase of up to 100-fold in *AANAT* mRNA. An activation of α1 receptors increases the activity of phospholipase C (PLC), which augments the intracellular calcium concentration ([Ca^2+^]_i_) that activates protein kinase C (PKC). This NE/α1/PLC/[Ca^2+^]_i_/PKC pathway per se does not directly stimulate AANAT or melatonin production, but it potentiates the β1 receptor activity to further increase AANAT, presumably via a calmodulin kinase.

**Figure 2:**
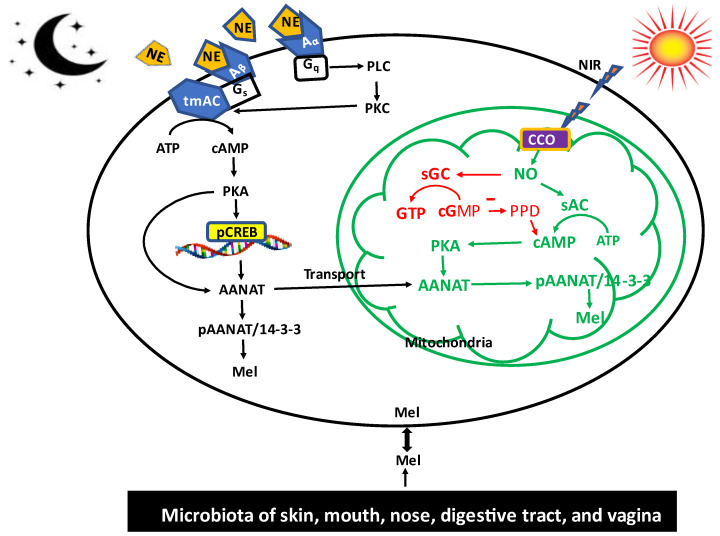
The differentially regulated melatonin synthetic pathway in cytosol, mitochondria, and microbiota. In the pineal, NE, which represents the dark message, acts on the Aβ and Aα membrane receptors to activate the tmAC to generate cytosolic cAMP, which is mitochondria impermeable. NIR is associated with sun exposure and acts mainly on the mitochondrial sAC to increase the mitochondrial cAMP, which in situ phosphorylates the AANTT at the posttranslational level. This model assumed an activation of sAC by NO, which would require further confirmation. In addition, NO activates sGC to increase cGMP, which can inhibit the phosphodiesterase to reduce cAMP degradation and further increases the mitochondria cAMP content. The posttranslational regulation of AANAT activity quickly increases the melatonin synthesis, as seen in extrapineal cells. The regulatory mechanisms of melatonin synthesis in microbiota are currently unknown, but it is hypothesized there is a reciprocal effect between the melatonin produced by the host and microbiota. NE: norepinephrine, Aβ: β adrenergic receptor, Aα: α adrenergic receptor, G_s_: stimulatory G protein, G_q_: G protein q subunit, tmAC: transmembrane adenylyl cyclase, sAC: soluble adenylyl cyclase, sGC: soluble Guanyl cyclase, PPD: phosphodiesterase, PLC: phospholipase C, PKC: protein kinase C, PKA: protein kinase A, AANAT: arylalkylamine acetyltransferase, pAANAT: phosphorylated AANAT, NO: nitric oxide, CCO: cytochrome C oxidase (mitochondrial complex IV), 14-3-3: 14-3-3 protein, Mel: melatonin.

In ungulates or primates, the pineal melatonin regulatory mechanisms are quite different from those of rodents. In the former, the pinealocytes maintain relatively high levels of *AANAT* mRNA without significant circadian alterations. However, AANAT protein monomers are catalytically inactive and subjected to rapid proteasomal degradation. In primates and ungulates, AANAT protein stability and activity is achieved by PKA- and PKC-catalyzed phosphorylation and the formation of a moderately stabilizing complex with 14-3-3ζ protein [111]. The limited stability of this complex allows for disassembly and dephosphorylation. As a consequence, AANAT is rapidly degraded, as soon as PKA and PKC are no longer activated because of decreasing cAMP and calcium levels. In rodents, the circadian rhythmicity of melatonin formation is regulated differently. Apart from the direct binding of pCREB to the AANAT promoter, the pineal circadian oscillator also activates this gene via the binding of BMAL1/CLOCK or BMAL1/MOP4 heterodimers to E-boxes in the promotor. Moreover, the activation of the *AANAT* gene is shut down, with a delay, by pCREB-mediated activation of the *ICER* (inducible cAMP early repressor) gene. The ICER protein suppresses *AANAT* expression via binding to the promoter. These regulatory molecular mechanisms have been extensively reviewed [112,113]. More details including modulation by other neurotransmitters and hnRNP complexes that control *AANAT* mRNA degradation can be found in [113]. It seems that the pinealocytes maintain a great capacity to synthesize melatonin and this ability is only maximized during darkness with the availability of NE; conversely, the melatonin synthetic capacity of the pinealocytes is minimized by light perception at the level of the eyes with visible light exposure. It is a remarkable fact that the circadian up- and downregulation of AANAT has been achieved in nature by two entirely different mechanisms, a transcriptional and a posttranslational one, however, with an identical initiator, NE release (Figure 2).

It is well-documented that blue light at wavelengths around 480 nm, which activates melanopsin in intrinsic photosensitive retinal ganglion cells (ipRGCs), is the most effective means of suppressing pineal melatonin biosynthesis [114,115]. The day/night alterations of the pineal melatonin production reflected by the CSF and serum melatonin circadian rhythms correspond to the natural photoperiodic conditions. Many vertebrates use these melatonin signals to adjust both daily and annual physiological activities such as hibernation and seasonal reproduction. Pinealectomy or light exposure at night disrupts these physiological activities and causes unfavorable biological consequences [116,117]. Since darkness promotes and blue light suppresses melatonin synthesis in pinealocytes, the high levels of melatonin in CSF and blood are always associated with the dark. Thus, melatonin is referred to as the chemical expression of darkness [11]. However, this expression now requires modification due to recent advancements in melatonin research. In addition to pinealocytes, perhaps all other cells synthesize melatonin and this extrapineal melatonin production is not generally suppressed by light and often lacks a circadian rhythm. An example of low-amplitude circadian rhythmicity in extrapineal melatonin formation would be the gastrointestinal tract, which was shown to peak somewhat earlier than the pineal-derived maximum [118,119,120]. Additionally, non-visible NIR may promote extrapineal melatonin production due to its unique optical ability to interact with various cellular molecules. For example, melanopsin was recently identified in fat cells [121] that are abundant in the hypodermis; fat cells could be the source of melatonin in the sweat of individuals exposed to sunlight. However, how melanopsin activation in fat cells by blue wavelengths would mediate a change in melatonin production in these cells has never been considered and whether NIR light would be relevant to this response would be mere conjecture. Considering the huge size of potentially melatonin synthetic tissues and organs compared to the pineal gland, they likely generate far more melatonin than the pineal gland and they do so even when individuals are exposed to sunlight, which contains NIR radiation.

## 4. Role of Non-Visible Near Infrared (NIR) Radiation on Melatonin Synthesis: Melatonin as a Participant of Sunlight Exposure

The most powerful environmental factor to impact pineal and retinal melatonin synthesis in the majority of the species is light, both natural sunlight and manufactured light. Sunlight provides up to 30 MJ/day to the body and represents the single largest energy input. Optically, the red/NIR portion of sunlight uniquely interacts with virtually all of our cells based on the biological windows between 650 nm and 1200 nm. NIR light penetrates the body to a depth of several inches [122]. As discussed by Zimmerman and Reiter [122], deep red and NIR are uniquely optically collected and travel through the CSF, amniotic fluid, and blood vessel walls, thereby stimulating a wide range of bio-optical effects that form the basis of most photobiomodulation (PBM) and low level light therapies (LLLT). The failure to understand the biological roles of light on our body has led to a static modern lifestyle, especially in children [122]. Incandescent sources introduced in the 1800s emitted 90% of their energy in the NIR. Starting in the 1950s, CFL and more recently LED lighting and displays, provide zero NIR. Coupled with Low E glass that blocks NIR from entering our homes, we now spend 93% of our time indoors exposed to zero NIR. This represents the largest reduction to solar exposure in human history. It is important that we understand the impact of eliminating 90% of the solar spectrum, which has had effects on biological processes such as melatonin production. Simultaneously, heavily modulated artificial light at night not found in nature has been introduced into the human environment. This has likely resulted in the increase in a wide range of diseases. The central role that melatonin plays in how the body responds to this new artificial environment deserves serious consideration. To reiterate, humans spend up to 93% of their time under artificial lighting and in front of displays that only emit between 400 nm and 700 nm, providing zero NIR. Initially, circadian theory suggests that the increase in blue light during the night disrupts sleep patterns. More recently, the focus has shifted toward getting more bright visible light exposure in the morning, causing the introduction of LEDs with an extra 480 nm emission. This, however, ignores 90% of the solar spectrum that has been removed from our homes, offices, and school. This radical change in light exposure correlates with the myopia epidemic, sleep disruption, increased breast cancer rates, reduced cognitive learning in children, and increases in a host of autoimmune diseases [123,124,125].

The regulatory mechanisms of light on melatonin biosynthesis in the pineal gland have been well-documented. These mechanisms, however, only apply to effects of the visible light (400 nm to 700 nm), particularly, the blue wavelengths, on the specific melatonin production in the pineal gland. Humans are exposed to solar spectral wavelengths ranging from 250 nm to over 4000 nm with the blue light only representing approximately 2% of the spectrum emitted by the sun. Blue light is strongly absorbed by our surroundings, and penetrates less than a millimeter into our skin. In contrast, NIR represents the majority of the solar spectrum, is strongly reflected by our surrounding, is optically collected, and uniquely interacts with a large percentage of the body’s cells, especially in children.

In excess of 70% of the photons impinging on the body from natural sunlight are NIR photons [122]. NIR is invisible and includes wavelengths from 650 nm to 1200 nm. Unlike visible light, which does penetrate the surface of the body, NIR can penetrate the skin and into the underlying tissues (e.g., muscles, blood, and even the brain) [126,127]. For example, the CSF in the subarachnoid space surrounding the brain optically functions as a guide and distributes NIR photons to neural tissue including that around the deep folds of the brain [122].

The beneficial effects of NIR on human health have been extensively studied. NIR therapy is collectively termed “photobiomodulation” (PBM). PBM has been applied clinically in the treatment of retinitis pigmentosa, age-related macular degeneration, soft tissue injuries, acceleration of wound healing, and recently, to Alzheimer’s disease [128,129,130]. Several mechanisms have been hypothesized for the therapeutic effects of PBM [131,132]. The most widely accepted one is an NIR/mitochondria interaction (i.e., the main target of NIR is mitochondria). When the NIR penetrates into the mitochondria, the potential mitochondrial photoreceptor molecule, cytochrome c oxidase (CCO), accepts NIR photons culminating in improved mitochondrial energy metabolism, increased cytoprotective factor production, and cell survival [129,133]. However, the detailed downstream molecular signaling pathways remain to be clarified. Since melatonin is synthesized in mitochondria and it is also a mitochondrially targeted antioxidant and protector, the effects of NIR have been linked to melatonin synthesis [134]; this speculation has been discussed in a recent report by Zimmerman and Reiter [121], who believe that some of the beneficial effects of NIR radiation on humans are mediated by increased melatonin synthesis in mitochondria. Indeed, many biophysiological outcomes of NIR treatment of animals or cultured cell lines are similar to those observed following melatonin treatment. These include that both treatments reduce ROS levels, preserve the mitochondrial membrane potential, inhibit the mitochondria permeability transition pore opening, balance mitochondrial dynamics, increase ATP production, and improve mitochondrial homeostasis [135,136].

Due to the similarity between NIR and melatonin interventions, we suggest a potential signaling pathway by which NIR illumination impacts melatonin synthesis at the cellular level. This speculated pathway is illustrated in Figure 2. Briefly, when the cells are exposed to the NIR of sunlight, the NIR photons penetrate cells and reach mitochondria. Cytochrome C oxidase (CCO) in the electron transportation chain of mitochondria (mitochondrial complex IV) is the primary photoreceptor. After absorbing the NIR photons, the excited CCO undergoes a conformational change that is believed to favor the release of the non-covalently bound nitric oxide (NO) at the heme and Cu centers of CCO into the mitochondrial matrix [137,138]. Whether CCO only releases NO formed by other enzymes may not be certain, since CCO was also discussed as an NO synthesizing enzyme due to its nitrite reductase activity [139]. While excessive NO causes oxidative stress, moderate levels of NO serve as a signaling molecule. It is our hypothesis that the NO released from the CCO promotes melatonin synthesis through the following pathway. The primary pathway is related to the enhanced activity of soluble adenylyl cyclase (sAC), which differs from tmAC and is primarily but not exclusively localized in the mitochondria [140,141]. sAC is known to be activated by bicarbonate and Ca^2+^ [133,142,143]. A relationship between sAC and NO would be of great interest. Some splice variants have been shown to carry a heme group that specifically binds NO, but not O_2_ [137]. Therefore, it seems to be an attractive idea to assume an activation mechanism by NO, similar to that in soluble guanylyl cyclase. The direct evidence for mitochondrial sAC activation by NO is still missing. In the study by Middelhaufe et al. [137], the authors were unable to increase the sAC activity by means of an NO donor. This negative result may have been caused by experimental difficulties and should be repeated using different approaches. However, the close association between NO release and sAC activation has been reported by Sisson et al. [144]. The sAC/cAMP/PKA pathway in mitochondria is well-established [145,146,147]. Since AANAT is present in the mitochondria, AANAT will be phosphorylated by activated PKA present in the mitochondria under NIR radiation. With high likelihood, pAANAT stabilized by recruited 14-3-3 would increase mitochondrial melatonin synthesis.

If validated, this posttranslational regulatory activity of AANAT in mitochondria by NIR may be very important. It would more rapidly enhance melatonin production compared to transcriptional regulation and thus would provide immediate protection to minimize cellular injury. This pathway may be identified as a short loop of NIR-induced melatonin synthesis.

Nevertheless, a more general relationship between NO and cAMP seems to exist, although this may not necessarily involve mitochondria. Various studies have found NO-mediated cAMP formation in different tissues and cells [148,149], but the molecular pathway has not been sufficiently clarified. Recently, it has been reported that in cilia, NO associated cAMP formation is not mediated by the classic tmAC, but rather by the NO/sAC/cAMP pathway [144]. In the cilia, NO also stimulated in parallel soluble guanylyl cyclase and led to cGMP mediated activation of PKG [136] (i.e., a classic pathway originally identified in smooth muscle relaxation) [150,151]. It has been reported that the NO activated sGC/cGMP pathway can also increase the cAMP content of cells by cross-talking between the cAMP and cGMP signaling pathways [149,152,153]. cGMP probably inhibits the phosphodiesterase to reduce cAMP degradation and thereby increases its concentration [154]. Therefore, the NO/sGC/cGMP/cAMP pathway also has the potential to increase melatonin synthesis in response to NIR.

Other processes may also contribute to NIR-induced melatonin synthesis. These include ROS induced melatonin synthesis. Exposure of cells to NIR increases ROS production. Low levels of ROS serve as signaling molecules, triggering NF-κB translocation into the nucleus, which upregulates AANAT expression. Increased expression of AANAT has been reported in oxidatively stressed pancreatic cells [155]. ROS induced melatonin synthesis in different tissues may be a response that prepares cells for upcoming catastrophic oxidative stress; without this preconditioning, cells may not survive high levels of free radical generation. The NIR exposure accompanied by enhanced melatonin production has been reported in different species. In apple tree leaves, an increased NIR exposure with seasonal changes in the photoperiod upregulates *SNAT* and *ASMT* (*HIOMT*) expressions and increases melatonin production [156]. In piglets, exposure to red-infrared heat lamp (597–780 nm) significantly increased their salivary melatonin level compared to exposure to heat lamp without NIR irradiation (ceramic lamp) [157]. The latter results indicate not the heat, but rather the NIR influenced melatonin synthesis. Zhao et al. reported that when young female athletes received whole body red light (658 nm and light dose of 30 J/cm^2^) irradiation for 30 min per night for 14 days, their morning (8:00 AM) serum melatonin was almost doubled compared to the control group [158]. In comparison, eight hours spent outdoors could deliver over 1000 J/cm^2^ of deep red and NIR into the body even through light clothing. Since red light has a weak impact on the retinal–pineal melatonin pathway and the eyes of the subjects were also covered with dark glasses, it was deduced that the increase in serum melatonin level was not of pineal origin but probably from the red light irradiated tissue such as skin.

**Figure 3:**
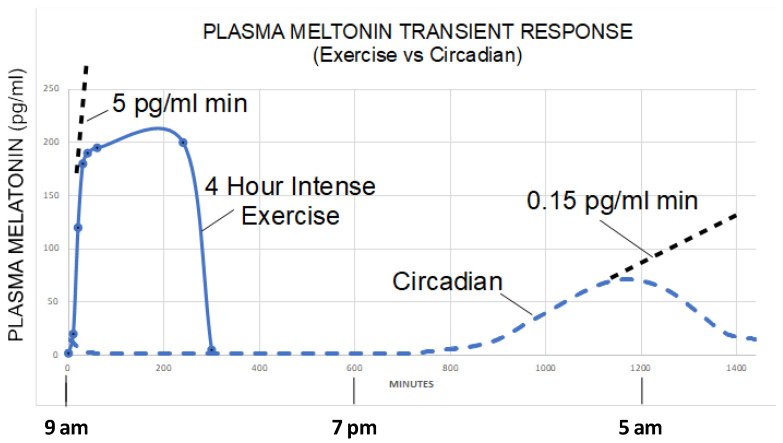
The plasma melatonin levels as a function of time during heavy exercise under sunlight and circadian time. During a 4-h intense exercise session, plasma melatonin levels rose to 200 pg/mL in 20 min followed by a plateau for the duration of the exercise (five test subjects with indwelling catheter measured plasma melatonin at 10, 20, 30, 40, 50, 60, 240, and 300 min, respectively). This figure was from [159] and permitted by the authors.

Recently, Zimmerman and Reiter [159] compared the real-time plasma data from Theron et al. [160] with the plasma and sweat data from Zhu [161] during strenuous exercise in artificial light and sunlight (Figure 3). The data support that large quantities of melatonin were being produced independent of the pineal gland; the data showed a greater than 5 pg/mL min ramp rate during sunlight exposure compared to a 0.15 pg/mL min ramp rate for plasma melatonin under dim light melatonin onset condition.

The data of Zhu [161] showed that sweat melatonin levels exhibited a low correlation with its associated plasma measurements, suggesting independent sources; however, the number of confounding variables limit conclusions that can be drawn. In general, the data supports the production of large amounts of melatonin in the skin and muscles during periods of high ROS generation, a response that deserves confirmation. Additional evidence may continue to emerge to show the association between ROS levels, exercise, NIR, and melatonin production. Thus, melatonin of pineal origin is the chemical expression of darkness in vertebrates, which is regulated by visible light as detected by the retinas. In comparison, extrapineal melatonin synthesis appears to be driven, in many tissues, by ROS levels at the cellular level with exercise and NIR promoting melatonin production in superficial cells or deep tissues. This may also be the case in reference to melatonin production by microbiota in the skin, mouth, nose, and vagina. The first two sites have the same melatonin synthetic enzyme system with different regulatory mechanisms; conversely, the microbiota have distinct melatonin synthetic genes, but the regulatory pathway has not been clarified.

## 5. Melatonin Synthetic System of the Gut Microbiota

Multi trillions of microbes inhabit the human skin, mouth, nose, digestive tract, and vagina. Mounting evidence shows that the relationship between these microbiomes and humans is not merely commensal, but is a reciprocal symbiosis [162]. Among these microbiota, those residing in the gut play a fundamental role in nutrition, metabolism, immunity, or even aging [163,164]. The microbiota are composed of bacteria, archaea, fungi, protozoa, and viruses. Many studies have documented that dysbiosis is associated with a variety of diseases and disorders including obesity, diabetes, autoimmune, and neurodegenerative diseases [165]. Interestingly, cross-talks between microbiota and mitochondria have been observed, in which ROS, nitric oxide, short chain fatty acids, and hydrogen sulfide act as communication signals [166]. A variety of studies have linked the regulatory effects of melatonin on gut microbiota and human health. For example, melatonin treatment reduces the obesity in mice by redistribution of their gut microbiota, particularly to decrease the Firmicutes-to-Bacteroidetes ratio and increase the abundance of mucin-degrading bacterium *Akkermansia*, which is associated with healthy mucosa [167]. Melatonin also improves lipid dysmetabolism in high-fat diet-fed mice by the reprogramming of gut microbiota [168]. Melatonin attenuates the microbiotal dysbiosis of jejunum in short-term sleep deprived mice [169].

Most studies have addressed the importance of the host or exogenously supplied melatonin on the health of microbiota. However, none of them have addressed the significance of the melatonin generated by microbiota on the host’s health. Here, we hypothesize that microbiota can also synthesize melatonin, and it is another melatonin source contributing the host’s total melatonin pool and health of the host. It is known that bacteria have the capacity to synthesize melatonin. These include primitive photosynthetic bacteria [95,170] as well as *Eschericha coli* [171]. Melatonin has been detected in unicellular eukaryotes [172,173] and high levels of melatonin are present in the wine, which is synthesized by yeast [174,175]. Moreover, AANAT has been identified in and also cloned from archaea [4]. Many organisms belonging to such basal groups are components of gut microbiota. Therefore, there is no reason to doubt that gut microbiota is an additional source of the host’s melatonin pool.

The primary function of microbiota-generated melatonin is their protection from environmental insults, particularly free radicals. For example, recombinant *E. coli* with melatonin synthetic enzymes increase their melatonin production by 8-fold above that in the wild type; as a result, their survival rate increases 100-fold compared to the WT under aluminum stress [176]. Additionally, the microbiota-generated melatonin may also boost the host’s protection. One clue suggesting that melatonin from microbiota enters the system of the host relates to the extremely high levels of bile melatonin in human and also in other animals [177]. The melatonin concentrations in the bile of different species range from 2000 to 11,000 pg/mL, which is two to three orders of magnitude higher than those in day time serum. It is possible that the majority of the biliary melatonin is of microbiota origin, which gains access to the enterohepatic circulation and is subsequently released to the bile [178]. Unlike animals, the melatonin synthetic capacity of the microbiota is not limited by the availability of tryptophan since microorganisms can use carbon dioxide or glucose as the substrates to synthesize melatonin. Extremely high melatonin levels have been reported in some microorganisms [179].

Direct evidence on the melatonin synthetic capacity of the gut microbiota has recently emerged. Kovtun et al. [152], with the aid of 16S rRNA sequencing technology, identified AANAT in human microbiota, indicating the melatonin synthetic capacity of the gut microbes. In the in vivo study, Ouyang et al. [180] observed that melatonin was present in the rumen fluid of cows, where it exhibited a circadian rhythm. When the rumen fluid was cultured under in vitro conditions, the melatonin circadian rhythm persisted. The results suggest that the melatonin, even though it was rhythmic, was generated by the microbiota of the rumen rather than by the host cell. More recently, Liu et al. observed that methanogenic archaea are an additional source of melatonin in the bovine rumen and that melatonin suppresses methane emissions from the rumen (patent application #202210128296.x); this could have serious benefits for the environment. In general, the biological activities of melatonin from the microbiota have remained uninvestigated. Based on the known antioxidant, anti-inflammatory, and immunoregulatory activities of melatonin, the protective effects of this melatonin on the digestive tract of the host likely involves these actions. The microbiota-derived melatonin, except for some special situations, probably do not routinely contribute to circulating melatonin since pinealectomy causes the blood melatonin levels to be near zero. However, stress-induced microbiota dysbiosis did significantly lower the daytime serum melatonin levels while melatonin administration reversed dysbiosis [181]. It is likely that with advances in this field, the potentially reciprocal interactions of the host’s melatonin and the melatonin generated by the skin, mouth, nose, digestive tract, and vagina microbiota will be clarified (Figure 2).

## 6. Discussion

**Figure 4:**
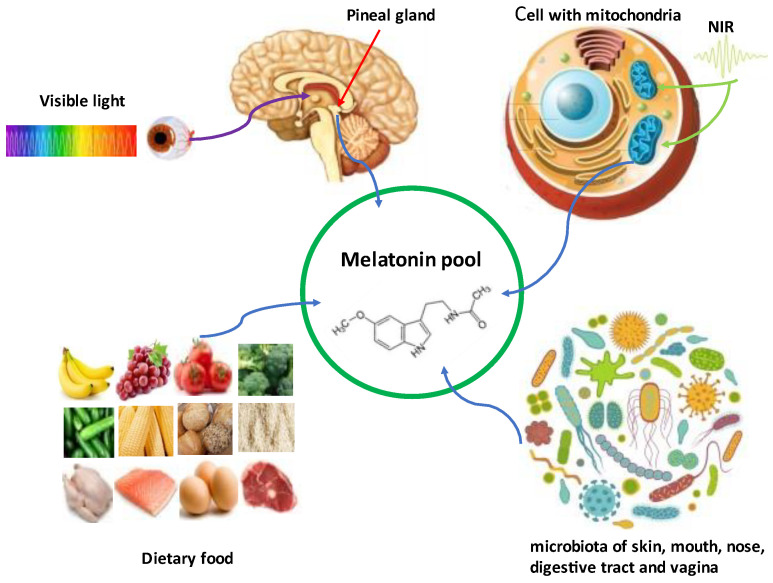
Illustration of the melatonin pool of vertebrates. NIR: near infrared radiation.

At least four differentially regulated melatonin sources contribute to the melatonin pool of animals including humans. These include (1) visible-light regulated melatonin of pineal origin; (2) NIR-regulated melatonin; (3) that originating from mitochondria as well as (4) dietary melatonin (Figure 4).

An additional source may exist in the microbiome. Visible light (blue light, especially at wavelengths around 480 nm) mainly acts in the regulation of retinal and pineal melatonin synthesis. The pineal-derived melatonin, which represents in higher vertebrates the major portion of the releasable pool, exhibits circadian and seasonal rhythms and serves as a signal of environmental photoperiodic changes [182]. Pineal melatonin is directly released into the third ventricle of the brain, which results in the extremely high melatonin levels in the CSF of the third ventricle compared to the blood melatonin concentrations. It is speculated that the alternations of CSF melatonin serve as the main light/dark signal that acts on the suprachiasmatic nucleus [106], while the blood melatonin is the residue of pineal melatonin not released to the third ventricle, which parallels the rhythm of CSF melatonin. The blood melatonin level can be modified by several means such as food intake [183], calorie restriction [184,185], meditation [186], exercise [187], etc. Changing the blood melatonin concentrations by these means does not significantly impact the extremely high CSF melatonin levels, and therefore will not disrupt CSF melatonin, which originated from the pineal gland as the day/night and seasonal signals.

Unlike visible electromagnetic radiation, NIR penetrates the skin, underlying tissues, and organs; thus it can directly impact the physiology of these cells. NIR radiation may increase local tissue mitochondrial melatonin production; this locally-produced melatonin has limited access to the blood. The locally-generated melatonin is used in its cell of origin to protect against oxidative stress and other insults. Theoretically, the NIR-induced total melatonin production may by far surpass the pineal-generated melatonin. For example, under sunlight, the skin and underlying tissues in response to NIR, their melatonin synthesis may increase many-fold compared to the dim light condition, judging by the sweat melatonin level [159]. Skin is the largest organ and, with the exception of a few areas, sweat glands are distributed throughout the dermis. When skin is exposed to the sunlight, the UV irradiation generates ROS and injures DNA and other macromolecules of the skin cells, which is associated with the increased incidence of melanoma. While the NIR simultaneously increases melatonin production to protect the cells from the UV damage, it does not interfere with the beneficial effect of UV on vitamin D biosynthesis. Moreover, the metabolites of melatonin also neutralize ROS; these include AFMK, AMK, and 6-hydroxymelatonin, which have been identified in epidermal cells [187,188].

Another important issue relates to the potential effects of NIR on the central nervous system, especially in diurnal animals such as humans. During the day when a diurnal species is active, the brain utilizes a disproportionally high level of oxygen, which increases ROS generation in neurons and associated cells. In addition, some other antioxidant systems are deficient in the brain [189]. This makes melatonin as a brain antioxidant particularly important. All brain cells including the neurons and astrocytes have the capacity to synthesize melatonin and the expression of melatonin synthetic enzymes has been identified in these brain cells [41,42]. In microglia, increases in melatonin formation may favor polarization to the anti-inflammatory type M2, as extracellular melatonin does [190]. Daily exposure to sunlight likely boosts the brain’s antioxidant capacity by increasing melatonin production due to the deep penetration of NIR into the brain through the guide of CSF.

We have also recognized that the differentially regulating mechanisms of melatonin synthesis between the pinealocytes and extrapineal cells are determined by receptors and where cAMP is involved. In pinealocytes, the increased release of NE by sympathetic nerve endings during darkness activates the tmAC and increases the cytosolic cAMP content. cAMP is mitochondrial membrane impermeable; thus, it can only activate the cytosolic PKA, and via the NE/Aβ1/tmAC/cAMP/PKA/CREB/AANAT transcriptional pathway, elevate melatonin synthesis, as documented in the rodent pineal gland. It may also enhance phosphorylation of the cytosolic AANAT by PKA, but due to the much lower substrate (acetyl CoA) availability than the Km of AANAT, the melatonin synthesis via this route is limited. In contrast, the NIR mainly activates the mitochondrial sAC to increase mitochondrial cAMP by posttranslational regulation of mitochondrial-located AANAT on-site. Therefore, it may quickly improve melatonin production. In addition, the NO released by NIR radiation can also defuse into the cytosol from mitochondria to activate cytosolic sAC to increase the cytosolic cAMP content, which elevates AANAT at either the transcriptional or the post-translational level, depending on the respective mammalian taxon. Thus, NIR-induced melatonin synthesis seems to be efficient and helpful in protecting the brain against oxidative damage during the day; conversely, at night, the active pineal gland generates high circulating melatonin concentrations in the CSF that take over this responsibility, a process that requires the newly-described movement of CSF through the glymphatic system [106]. In summary, the brain relies on the antioxidant activity of melatonin during both day and night, but the processes involved in the respective modes of protection are different. NIR exposure may also aid in the production of melatonin in other organs, especially those that are rich in mitochondria (e.g., brown adipose tissue, muscle, etc.). Obviously, melatonin is important under both sunlight conditions and darkness, even when the circulating melatonin values may be minimal. Thus, melatonin is an important physiological agent in both the day and night with regard to antioxidant protection.

The regulatory mechanisms of melatonin synthesis by microbiota are unknown. Preliminary data suggest that at least in the bovine rumen, melatonin synthesis may be rhythmic. If so, melatonin synthesis in microbiota might be driven by food consumption (gut microbiota), sunlight (skin microbiota), pH (vaginal microbiota), or as a consequence of yet-to-be-defined processes that will be clarified in future studies.

## Data Availability

Not applicable.
